# Identification of functional differences between recombinant human α and β cardiac myosin motors

**DOI:** 10.1007/s00018-012-0927-3

**Published:** 2012-02-16

**Authors:** John C. Deacon, Marieke J. Bloemink, Heresh Rezavandi, Michael A. Geeves, Leslie A. Leinwand

**Affiliations:** 1 Department of Molecular, Cellular and Developmental Biology and Biofrontiers Institute, University of Colorado, MCDB, UCB 347, Boulder, CO 80309 USA; 2 School of Biosciences, University of Kent, Canterbury, CT2 7NJ UK

**Keywords:** Muscle, Kinetics, ATPase, Contraction, Heart

## Abstract

The myosin isoform composition of the heart is dynamic in health and disease and has been shown to affect contractile velocity and force generation. While different mammalian species express different proportions of α and β myosin heavy chain, healthy human heart ventricles express these isoforms in a ratio of about 1:9 (α:β) while failing human ventricles express no detectable α-myosin. We report here fast-kinetic analysis of recombinant human α and β myosin heavy chain motor domains. This represents the first such analysis of any human muscle myosin motor and the first of α-myosin from any species. Our findings reveal substantial isoform differences in individual kinetic parameters, overall contractile character, and predicted cycle times. For these parameters, α-subfragment 1 (S1) is far more similar to adult fast skeletal muscle myosin isoforms than to the slow β isoform despite 91% sequence identity between the motor domains of α- and β-myosin. Among the features that differentiate α- from β-S1: the ATP hydrolysis step of α-S1 is ~ten-fold faster than β-S1, α-S1 exhibits ~five-fold weaker actin affinity than β-S1, and actin·α-S1 exhibits rapid ADP release, which is >ten-fold faster than ADP release for β-S1. Overall, the cycle times are ten-fold faster for α-S1 but the portion of time each myosin spends tightly bound to actin (the duty ratio) is similar. Sequence analysis points to regions that might underlie the basis for this finding.

**Electronic supplementary material:**

The online version of this article (doi:10.1007/s00018-012-0927-3) contains supplementary material, which is available to authorized users.

## Introduction

**Fig. 1:**
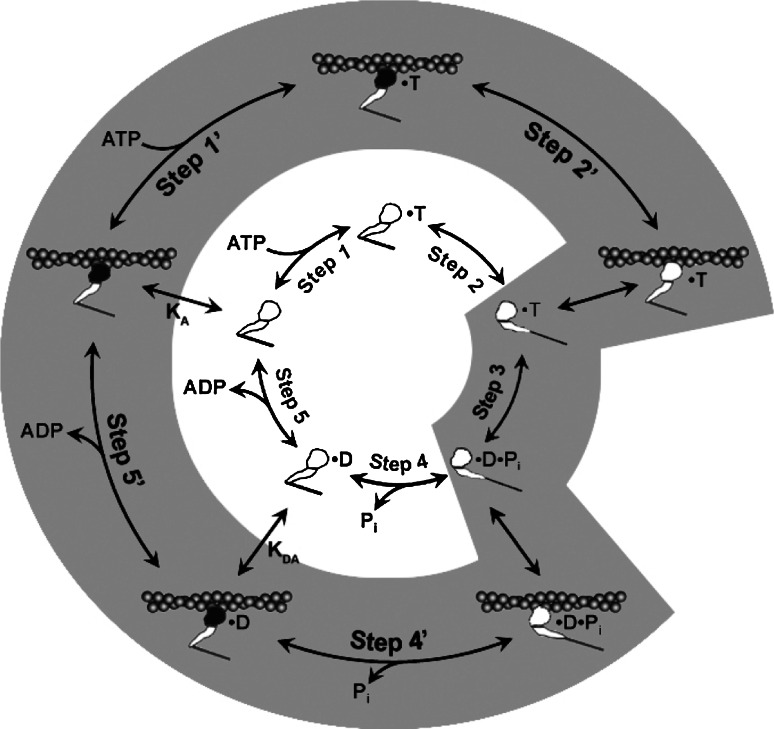
Myosin contractile cycle. Myosin motors shown graphically interacting with actin filaments and nucleotides as is modeled to occur in the contractile cycle. ATP, ADP, and phosphate are represented by T, D, and P_i_, respectively. Strong actin–myosin binding is indicated by *black motor domains* and low actin-affinity states by *white motor domains*. Steps occurring while bound to actin are indicated as step 1′–5′, and those while detached from actin as step 1–5. The *highlighted path* is the main active contractile cycle. Steps 1 and 1′ are dependent upon the equilibrium constants of ATP binding *K*_1_ and *K*′_1_, respectively. Steps 2 and 2′ are dependent upon the rate constants of a conformational change in the motor domain associated with loss of actin affinity *k*_+2_ and *k*′_+2_ respectively. Step 3 is dependent upon the rate constant of ATP hydrolysis *k*_+3_ + *k*_−3_. Steps 4 and 4′ are dependent upon the rate constants of P_i_ release *k*_+4_ and $k_{{ + 4}}^{\prime}$ respectively. Step 5 and 5′ are dependent upon the rate constants of ADP release *k*_−ADP_ and $k_{\rm{ -ADP}}^{\prime}$, respectively. Dissociation of myosin from actin in the absence of nucleotide is governed by the dissociation constant *K*_A_. Dissociation of myosin from actin in the presence of ADP is governed by the dissociation constant *K*_DA_. Dissociation of myosin from actin after Step 2′ is essentially diffusion-limited

Myosins are the molecular motors responsible for muscle contraction via the ATP-driven cross-bridge cycle, outlined in Fig. 1. Current interest in the myosin family of motors is focused on how this ATP-driven cross-bridge cycle is adapted for a wide range of different mechanochemical functions. Conventional myosins are the best-known family of motors, consisting of two heavy chains (MyHC) and two pairs of light chains: regulatory light chains (RLC) and essential light chains (ELC). The C-termini of the MyHCs dimerize and form a coiled-coil tail and the N-termini form the two myosin ‘heads’ or ‘motor-domains’. A lever arm, stabilized by binding of the ELC and RLC, transfers the conformational changes occurring in the motor domain into directional movement along the actin filament [1]. The single globular motor domain (often referred to as S1) is responsible for the motor function of myosin and contains the sites for both actin and nucleotide binding [2, 3].

Different isoforms of the myosin II family are found in striated, cardiac, and smooth muscle and non-muscle cells. The eight traditional striated muscle myosins II share 79% sequence identity in the motor domains whereas the identity within the fast skeletal and cardiac families is much higher. For example, there are two cardiac myosins α and β, which share 91% identity in their motor domains [4]. With such high sequence identity, the differences in behavior of the two cardiac isoforms might be expected to be modest, and indeed studies of rodent cardiac myosin suggest that the actin-activated ATPase and in vitro motility velocities differ ~two- to three-fold [5]. Single molecule assays showed that the force per cross-bridge and the magnitude of the working stroke do not change significantly between the cardiac isoforms [6].

While there have been many biochemical kinetic analyses of total striated muscle myosins from individual muscles, these often contain mixed isoforms [7]. To understand the structure–function relationships among the sarcomeric myosins it is essential to work with pure single isoforms of myosin. This has been done for the few myosin isoforms that are predominantly expressed in a muscle tissue (e.g., chicken pectoral, >95% MyHC I/β; rabbit psoas, 92% MyHC IId/x; rabbit soleus 97% MyHC I/β and bovine masseter, predominantly MyHC I/β) [8, 9]. These studies showed that each myosin undergoes the same cross-bridge cycle (as outlined in Fig. 1) but each isoform has distinct rate and equilibrium constants for each step in addition to changes in the overall ATPase rates. An alternative approach has been to purify small amounts of myosin or S1 from single muscle fibers, which can be selected based on expression of a single isoform [10]. This allows direct correlation between mechanical properties of single muscle fibers with a limited number of assays made on myosin from the same fiber. Both types of studies support the hypothesis that the maximum shortening velocity of a muscle fiber is defined by the myosin isoform expressed in the fiber. Biochemically, shortening velocity is defined by the rate at which cross-bridges detach from actin limited by either the rate at which ATP binds to the cross-bridge (Step 1′ and 2′ in Fig. 1) or the preceding ADP release step, Step 5′ [10, 11]. While there have been a number of steady-state measurements of actin-activated ATPase from a variety of myosin isoforms [12, 13], there have been relatively few detailed kinetic studies of mammalian skeletal and cardiac myosins.

Mammalian hearts express α and β MyHCs from two separate genes. The ratio of expressed isoforms varies between species and also within species at various stages of development and between atrium and ventricle (for a review, see [14]). Larger mammals express predominantly β-MyHC in their cardiac ventricles (hereafter referred to as hearts) while hearts of small mammals such as rats and mice express predominantly α-MyHC. It is also known that many different stimuli (disease, exercise, hormonal status) can affect the relative proportions of the two cardiac isoforms [15, 16]. For example, in rodent hearts, induction of β-MyHC is a hallmark of pathology. Work from our laboratory and others has demonstrated that healthy human hearts express ~10% of their myosin as the α-MyHC isoform. In contrast, failing human hearts express no detectable α-MyHC [15, 16]. Significantly, α-MyHC is re-expressed after pharmacologic intervention only in patients with improved function [17]. Work in isolated myocytes has demonstrated that even small amounts of α-MyHC can increase power output [18]. Therefore, understanding the biochemical kinetic properties of α and β isoforms is an important first step in understanding the role these changes in isoform expression levels play in cardiac function in health and disease.

Kinetic studies on cardiac myosins have concentrated exclusively on the β isoform, which is the predominant isoform in the ventricles of large mammals and it is also expressed in slow skeletal muscle fibers. The β isoform has been of special interest because of its prominent role in a series of familial cardiomyopathies [19]. There are no published studies of the kinetics of an α isoform from any species. The difficulty of isolating pure human myosin for a detailed biochemical characterization has recently been overcome by the expression of human muscle MyHCs in mammalian muscle cells [13]. Winkelmann et al. [20] pioneered the use of the mammalian skeletal C_2_C_12_ muscle cell line to examine the role of chaperones in proper folding of the motor domain of sarcomeric myosins. This approach was first used by Wang et al*.* [21] to express embryonic chicken myosin and to measure the in vitro motility of the isolated myosin. Based on these studies, we developed a C_2_C_12_-based expression system that produces soluble and active recombinant *human* striated muscle myosin motors [13]. We expressed proteins containing the globular motor domain and the IQ domains involved in binding both ELC and RLC, analogous to papain-derived S1.

Here we describe the primary events in the actin–myosin ATPase cycle for human α- and β-S1 as a first step in defining the steps in the cycle that contribute to their functions. These data are compared to data on mouse α-S1 purified from cardiac muscle and our published data on tissue-purified β-S1 from the cow, rabbit, and pig. Surprisingly, given the high degree of identity between α and β human myosins, several events in the cross-bridge cycle differ by as much as ten-fold. These include the ATP hydrolysis step, which controls the lifetime of the detached myosin and the ADP release from actin·S1.

## Experimental procedures

### Motor domain constructs

PCR products amplified from human heart cDNA were cloned into the MCS of pShuttle-CMV modified to seamlessly add a 6xHistidine tag in frame with the C-terminus of S1, where an AflII site was silently coded into the conserved S1 C-terminal amino acid sequence *LK*SA. Translation is terminated after this sequence in S1 such that it includes Met1-Ala843. The respective α protein terminates at the same conserved amino acid, but due to sequence differences is slightly longer, including Met1-Ala845. Unmodified pShuttle-CMV was used in cloning β short-S1 (β-sS1) where no tag was added and translation terminated after the ELC-binding IQ domain at Arg808. The human ventricular ELC, MYL3, was cloned into unmodified pShuttle-CMV and an N-terminal 6xHistidine tag was added by sequential elongating PCR steps.

### Adenovirus production

Adenovirus production was performed using the AdEasy kit (Qbiogene) with modifications [13]. Briefly, after cloning each construct into pShuttle-CMV, the shuttle vector was linearized with PmeI and homologously recombined with pAdEasy in bacteria. Successfully recombined plasmids were linearized with PacI and transfected into HEK293 cells stably expressing the E1 protein to complement pAdEasy for replication competence. Infected cell lysates were used to infect increasing numbers of cells, then virus isolated from the lysates by sequential step and linear CsCl gradients. Purified virus was stored at −20°C in 100 mM Tris pH 7.5, 250 mM NaCl, 1 mM MgCl_2_, 1 mg/ml BSA, 50% glycerol.

### Cell culture and protein expression

Murine C_2_C_12_ myoblasts (ATCC) were cultured in Dulbecco’s modified Eagle’s Medium (DMEM) (Gibco) with 10% fetal bovine serum (FBS) (Hyclone). Cells were differentiated into myotubes at ~100% confluence in 2% horse serum media (Gibco-BRL). Forty-eight hours later, myotubes were infected with 1 × 10^6^–1 × 10^8^ plaque-forming units of adenovirus per 100 mm plate. Approximately 4 days after infection, the cells were collected in 250 μl per plate of lysis buffer (20 mM Imidazole pH 7.0, 100 mM NaCl, 50 mM Tris, 3 mM ATP, 0.5% Tween-20, 1 mM DTT, 1× Complete, EDTA-free protease inhibitor cocktail [Roche]) and lysed using a Dounce homogenizer. Lysates were clarified by centrifugation in a Beckman 50 Ti rotor at 30 kRPM for 30 min. Clarified lysates were run through 1-ml HisTrap HP columns (GE Healthcare) by FPLC using an AKTA Purifier (GE Healthcare). Single peak fractions were isolated by 300 mM Imidazole step gradient elution. After dialysis into 25 mM Imidazole pH 7.0, 10 mM KCl, 4 mM MgCl_2_, 1 mM DTT, eluates were further purified by FPLC through 1-ml HiTrapQ HP columns (GE Healthcare). Single peak fractions were isolated at ~300 mM NaCl by a 20-column volume linear gradient elution. Purified proteins were stored at 4°C on ice and dialyzed into the appropriate reaction buffers before use.

### Proteins derived from muscles

Cardiac myosin was prepared from a heart of an adult mouse (CD1) according to Margossian and Lowey [22]. S1 was prepared from myosin by digestion with chymotrypsin as described by Weeds and Taylor [23]. The mouse α-S1 was not stable for more than few days on ice and was therefore stored in the presence of an EDTA-free protease inhibitor cocktail (Roche), and used without extensive purification within 48 h of digestion by chymotrypsin. Actin was prepared from rabbit muscle as described by Spudich and Watt [24] and labeled with pyrene iodoacetamide as described by Criddle et al. [25].

### Transient kinetics

Quenched-flow experiments were performed using a Hi-Tech RQF-63. All measurements were made using a buffer containing 20 mM MOPS pH 7.0, 100 mM KCl, 5 mM MgCl_2_. S1 was mixed with ten-fold excess ATP and incubated at 20°C for various time points (10–500 ms), then quenched by 1:1 addition of 6.25% (w/v) trichloroacetic acid. After neutralization with NaOH and a clarification spin at 3,000 ×* g* for 5 min, ADP and ATP were separated for each time point by HPLC using a Hypersil ODS (3 μm) column by isocratic flow, and quantified by integration of peak areas. For each time point, the ratio of ADP over total nucleotide concentration was used to calculate the hydrolysis rate constant for S1.

Stopped-flow experiments were performed essentially as described previously [26] using a HiTech Scientific SF-61DX2 stopped flow system. All measurements were made at 20°C (unless indicated otherwise) in 20 mM MOPS pH 7.0, 100 mM KCl, 5 mM MgCl_2_, 1 mM DTT. Fluorescence transients were measured using intrinsic S1 tryptophan fluorescence (excitation at 295 nm, emission through a WG320 filter) or pyrene-labeled actin (excitation 365, emission through a KV389 filter) [26]. Fluorescence transients were fitted to single or double exponential equations:

1 $$ F_{t} = {\text{Amp}}\cdot{\text{e}}^{{( - k_{\text{obs}} \cdot{t})}} + F_{\infty } $$

or

2 $$ F_{t} = {\text{Amp}}_{(1)} \cdot{\text{e}}^{{( - k_{{{\text{obs}}(1)}} \cdot{t})}} + {\text{Amp}}_{(2)} \cdot{\text{e}}^{{( - k_{{{\text{obs}}(2)}} \cdot{t})}} + F_{\infty } $$

and the fitted values of *k*_obs_ and the amplitudes were used to deduce rate and equilibrium constants of the events in the cross-bridge cycle as defined in Fig. 1. *k*_obs_ values were plotted against the varied nucleotide or S1 concentrations and fitted to the appropriate equation. Concentration in the text and figures refer to the concentration after mixing 1:1 unless otherwise stated.

**Scheme 1:**
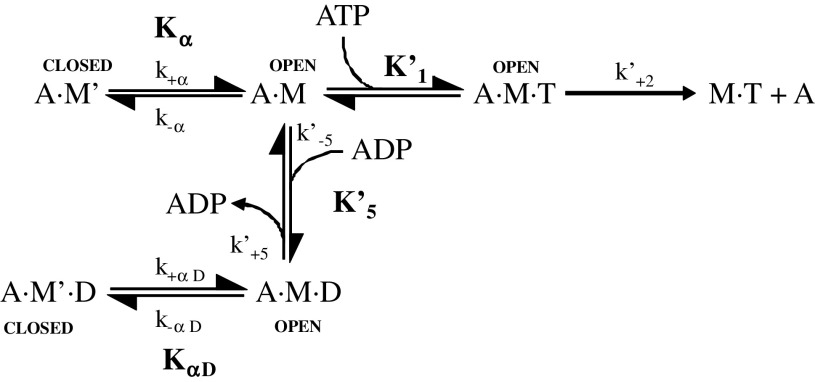

The outline of the ATPase cycle is shown in Fig. 1. In each case, the steps in the cycle are known to be more complex. For example, ATP binding, ADP release, and P_i_ release each consist of at least two steps: a diffusion-limited fast equilibrium step and a protein isomerization or conformational change. Similarly, the ATP hydrolysis step consists of at least two steps: the closure of switch 2 and the associated lever arm swing, which is known as the recovery stroke, occurs before ATP cleavage into ADP and P_i_. However, the simpler outline of Fig. 1 is sufficient for most of the data presented here except as outlined below in Scheme 1.

In Scheme 1, T and D represent ATP and ADP and where actin·S1 exists in two conformations (A·M and A·M′) in equilibrium. A·M′ is unable to bind nucleotide and must isomerize to A·M before ATP can bind. A similar pair of conformations exist in the presence of bound ADP, where A·M′·D must isomerize to A·M·D before ADP can dissociate.

For Figs. 3b, 4d, and 6b, the dependence of *k*_obs_ on ATP concentration is defined by:

3 $$ k_{\text{obs}} = k_{ \max } \left[ {\text{ATP}} \right] /\left( {K_{ 0. 5}   { + }\left[ {\text{ATP}} \right]} \right) $$

where *k*_max_ is the maximum value of *k*_obs_ and *K*_0.5_ is the nucleotide concentration required to give *k*_obs_ = *k*_max_/2.

For the ATP dissociation of A·M in Fig. 4a, d $K_{\rm max} = K^{\prime}_{+2}$, and $K_{\rm max} = K^{\prime}_{1}$ *K*_0.5_ = 1/*K*′. For Fig. 6b the ATP binding to S1, the ATP binding is considered to be irreversible and the data is not expected to conform to a hyperbola. In this case the fit is used to estimate the value of *k*_max_, which can be either *k*_+2_ or *k*_+3_ + *k*_−3_ and the initial slope of the plot defines *K*_1_ *k*_+2_.

For Fig. 4a the competitive inhibition of ATP induced dissociation by ADP can be described by $K_{{{\rm{obs}}}}  = K^{\prime}_{1} K^{\prime}_{{ + 2}} [ - {\rm{ATP]/(1 + }}K^{\prime}_{1} [{\rm{ATP] + [ADP]/}}K^{\prime}_{5}.$

If [ATP] is low, i.e., $K^{\prime}_{1}[\rm ATP] \ll 1$ then the above equation simplifies to: $K_{{{\rm{obs}}}}  = K^{\prime}_{1} K^{\prime}_{{ + 2}} [ {\rm{ATP]/(1 + [ADP]/}}K^{\prime}_{5})$ and this is rewritten as: $K_{{{\rm{obs}}}} /K_{{\rm{o}}}  = K_{{{\rm{obs}}}} K^{\prime}_{1} K^{\prime}_{{ + 2}} [ - {\rm{ATP] = 1/(1 + [ADP]/}}K^{\prime}_{5} )$ where *k*_o_ = the value of *k*_obs_ in the absence of ADP and at a fixed low concentration of ATP [11]. This assumes that ADP is in rapid equilibrium with A·M on the time scale of the measurement.

For Fig. 5 the analysis of the titration of S1 binding to actin was performed as described by Kurzawa and Geeves [27]. Using a fixed concentration of pyrene-actin and increasing S1 concentrations, the concentration of the actin·S1 complex can be estimated from the amplitude of the observed fluorescence transient when ATP is added to dissociate the complex. The amplitude dependence on [S1] data was then fitted to the physically significant root of the following quadratic equation.

4 $$ {{\upalpha}} = \frac{{ [M ]+ K_{\text{D}} + [A ]_{ 0} - \sqrt {\left( {[M] + K_{\text{D}} + [A]_{0} } \right)^{2} - \frac{4}{{ [M ] [A ]_{ 0} }}} }}{{ 2 [A ]_{ 0} }} $$

α is the fraction of actin with myosin bound, [*M*] is the total concentration of S1 added, [*A*]_0_ is the concentration of actin and *K*_D_ is the dissociation constant of S1 for actin (i.e., *K*_A_ or *K*_DA_).

For Fig. 6d, the two amplitudes of the ADP displacement reaction are proportional to the concentration of the S1 present as free S1 or S1·ADP. The amplitudes depend on the total ADP concentration as defined by:

5A $$ A   { = }\left( {A_{ \max } \left[ {\text{ADP}} \right]} \right) /\left( {K_{ 5}   { + }\left[ {\text{ADP}} \right]} \right)   { + }A_{ \min } $$

and

5B $$ A   { = }\left( {A_{ \max } K_{ 5} } \right) /\left( {\left[ {\text{ADP}} \right]   { + }K_{ 5} } \right)   + A_{ \min } $$

In all cases, the figures refer to individual experimental measurements whereas Table 1 gives the mean values of the fitted constant for 2–3 separate measurements.

**Table 1:** Comparison of rate and equilibrium constants for α-and β-S1

	Rabbit skeletal S1^b^	Mouse cardiac α-S1^a^	Human cardiac α-S1^a^	Human cardiac β-S1^a^	Human cardiac β-sS1^a^	Bovine masseter β-S1^c^
ATP-binding to S1						
*K*_1_ *k*_+2_ (μM^−1^s^−1^)	1.9	1.4	2.2 ± 0.1	1.5 ± 0.3	1.5 ± 0.1	0.97
*k*_+2_ (s^−1^)	>1,000^d^	>200	>200	158 ± 18	160 ± 23	117
*k*_+3_+*k*_−3_ (s^−1^)	131	150	168 ± 28	17 ± 2	14	18
ADP-binding to S1						
*K*_5_ (μM)	2	2.8	2.8 ± 0.7	0.5 ± 0.1	1.0 ± 0.1	2.0
*k*_+5_ (s^−1^)	1.4	3.5	2.7 ± 0.6	0.9 ± 0.1	0.59 ± 0.08	1.0
ATP-binding to actin·S1						
$k_{1}^{\prime} k_{{ + 2}}^{\prime}$ (μM^−1 ^s^−1^)	2.4	2.0	2.5 ± 0.3	1.1 ± 0.1	1.6 ± 0.3	1.24
1/$k_{1}^{\prime}$ (μM)	520^e^	216	626 ± 143	1,140 ± 65	710 ± 65	984
$k_{+2}^{\prime}$( s^−1^)	1,250^e^	>1,000	1,500 ± 167	1,445 ± 150	1,081 ± 50	1,220
*K*_α1_	*	10	10 ± 2	8 ± 2	12 ± 2	7.7
*k*_+α1_ (s^−1^)	*	47	78 ± 30	56 ± 12	49 ± 5	56
ADP-affinity for actin·S1						
$k_{5}^{\prime}$(μM)	120	250	152 ± 25	21 ± 3	10 ± 3	9.6
$k_{+5}^{\prime}$(s^−1^)	>1,200	>1,000	>1,252	93 ± 5	64 ± 3	94
*K*_αD_	*	*	*	7 ± 1	3.7 ± 0.4	5.3
*k*_+αD_ (s^−1^)	*	*	*	15 ± 0.6	1.8 ± 0.1	9.6
S1-affinity for actin						
*K*_A_ (nM)	33	25	37 ± 11	8 ± 2	17 ± 7	7.0
* K*_DA_ (nM)	1,000	nd	1,844 ± 546	191 ± 17	229 ± 138	37

Human α- and β-S1 constructs with mouse light chains or with human MYL3 (β-sS1) are compared to rabbit fast skeletal S1 and bovine masseter slow skeletal myosin S1 from muscle. The data presented here represent the mean values of 2–3 individual measurements. Measurements were performed at 20°C and 100 mM KCl, pH 7.0 unless indicated otherwise

*nd* not determined

^a^This study

^b^From Ritchie et al. [58] unless otherwise stated

^c^From Bloemink et al. [26]

^d^From Miller and Geeves [59]

^e^From Nyitrai et al. [10]. This work gives values of *k*_+2_ (= 740 s^−1^) and 1/*K*′_1_ (= 520 μM) at 12°C. The value of 1/$k_{1}^{\prime}$ is independent of temperature so the value of $k_{+2}^{\prime}$ at 20°C can be estimated from the $k_{1}^{\prime}$ $k_{+2}^{\prime}$ value at 20°C given in the table and 1/$k_{1}^{\prime}$, i.e., $k_{+2}^{\prime}$ = 2.4 × 520 = 1,248 s^−1^

No detectable slow component observed

## Results

### Recombinant human α- and β-S1 proteins

**Fig. 2:**
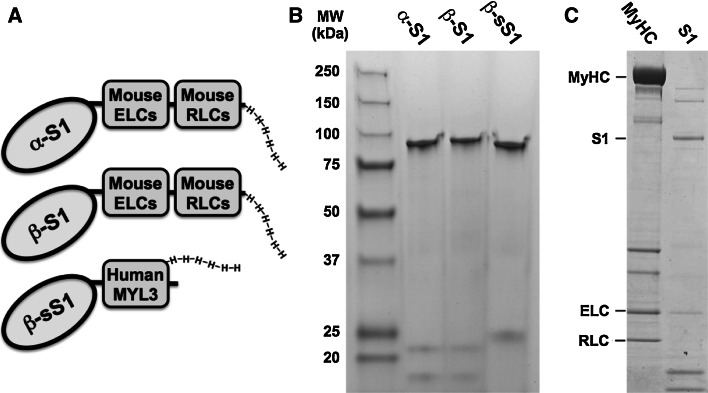
**a** Recombinant human α- and β-S1 proteins are C-terminally 6xHis affinity tagged for purification and copurify with C_2_C_12_ LCs. By coexpressing N-terminally affinity-tagged human ventricular ELC isoform MYL3 with untagged β-sS1 a humanized subfragment of MyHC can be purified. **b** SDS-PAGE of purified recombinant MyHC proteins. *Lane 1* contains Precision Plus Protein™ Dual Color Standards. ~98-kDa S1 proteins in *lanes 2* and *3* copurify with mouse ELC and RLC isoforms at <20 kDa and ~22 kDa. In *lane 3*, β-sS1 and MYL3 copurify at ~93 and 25 kDa, respectively. **c** SDS-PAGE of cardiac myosin and S1 purified from mouse heart. Full-length myosin (*1st lane*) copurifies with both ELC and RLC. Chymotryptic S1 purifies with only ELC

Using a previously described muscle cell expression system [13], we have produced recombinant human α- and β-S1 motors. S1 constructs encode binding domains for both ELC and RLC. A schematic diagram of each expression construct is shown in Fig. 2a. Adenoviruses encoding human α- and β-S1 were used to infect C_2_C_12_ myotubes and the protein was purified using the His-tag on the C-terminus of the motor. α-S1 and β-S1 co-purify with endogenous mouse ELC and RLC from the C_2_C_12_ muscle cells which were previously identified by mass spectrometry of the purified proteins as MLC1F, MLC2F, MLC3F, and MLC1A [13]. Because of a potential concern about using mouse light chains on a human motor, we also prepared a shorter β subfragment (β-sS1) corresponding to chymotryptic S1 [23] co-expressed with the human ventricular ELC isoform MYL3 and purified it using a 6xHistidine tag on the N-terminus of the ELC (Fig. 2a). Figure 2b shows a Coomassie-stained gel of the purified proteins.

### ATP-induced dissociation of actin·S1 is biphasic for human α- and β-S1

**Fig. 3:**
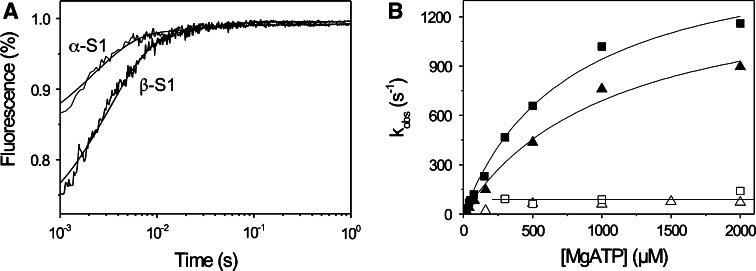
ATP-binding to actin·S1 (α- or β-isoform). The ATP-binding properties of the recombinant human α- and β-S1 proteins with actin present were investigated using stopped flow measurements. **a** After rapidly mixing 0.5 mM ATP with 0.1 μM pyrene-actin·S1, for both cardiac S1 isoforms the pyrene fluorescence transient is best described by a two exponential fit. For the fast phase, the observed rate constant was (*k*_obs_) = 658 s^−1^ (amp = 21%) for α and *k*_obs_ = 436 s^−1^ (amp = 31%) for β, whereas for the slow phase, a similar *k*_obs_ was found for both isoforms, *k*_obs_ = 61 s^−1^ (amp = 2.2%) for α and 64 s^−1^ (amp = 5%) for β. **b** For both α- and β-S1 the *k*_obs_ of the fast phase showed a hyperbolic dependence on ATP concentration. At high [ATP], *k*_obs_ for the fast phase (= $k_{+2}^{\prime}$ ) saturates at 1,667 for α (*filled square*) and at 1,432 s^−1^ for β (*filled triangle*) with a half maximal *k*_obs_ at 769 and 1,075 μM ATP (= 1/$k_{1}^{\prime}$) for α and β, respectively. The slower phase is virtually independent of ATP concentration *k*_+α1_ = 40–60 s^−1^ for both α (*open square*) and β (*open triangle*)

The steps in the cross-bridge cycle are shown in Fig. 1. The maximum shortening velocity of a muscle fiber is thought to be limited by how fast the myosin cross-bridge detaches from actin. This is limited either by the rate of ADP release from the cross-bridge (Step 5′) or the subsequent ATP-binding step (Step 1′ and 2′) [10, 11, 28]. The rate constants for these steps are a characteristic of the myosin isoform expressed in a muscle fiber [10]. For fast skeletal muscle myosins such as MyHC IIx from rabbit psoas, ADP release is very fast and detachment may be limited by how fast ATP binds to the cross-bridge [10]. For a slow muscle myosin, such as β-myosin, ADP dissociation is slow and limits how fast ATP can bind and thereby induce detachment [26, 29]. We measured the ATP-induced dissociation of actin·S1 for both human cardiac isoforms using stopped-flow methods as shown in Fig. 3. After rapid mixing of 0.5 mM ATP with 0.1 μM pyrene-actin·S1, for both cardiac S1 isoforms the pyrene fluorescence transient was best described by a two exponential fit with a large fast phase and smaller slow phase (Fig. 3a). For the fast phase, the observed rate constants for the two motors differed: *k*_obs_ = 658 s^−1^, amp = 21% for α and *k*_obs_ = 436 s^−1^, amp = 31% for β whereas for the slow phase a similar *k*_obs_ was found for both isoforms, *k*_obs_ = 61 s^−1^, amp = 2.2% for α and 64 s^−1^, amp = 5% for β. For both isoforms the *k*_obs_ of the fast phase showed a hyperbolic dependence on ATP concentration and saturates at high [ATP] with *k*_max_ = 1,667 and 1,432 s^−1^ for α and β respectively (see Fig. 3b) where *k*_max_ corresponds to $K^{\prime}_{+2}$ in Scheme 1. The values of *K*_0.5_ (= 1/$K^{\prime}_{1}$ *K*′_1_) were 769 μM for α and 1,075 μM for β-S1. The slow phase is virtually independent of ATP concentration and *k*_obs_ (= *k*_+α_) = 40–60 s^−1^ for both α- and β-S1. Table 1 contains the mean values for at least three measurements. The apparent second-order rate constant of ATP-binding ($K^{\prime}_{1}K^{\prime}_{+2}$) is about two-fold faster for α-S1 compared to β-S1. This is mainly caused by a two-fold tighter affinity of actin·α-S1 for ATP (1/$K^{\prime}_{1}$) compared to β-S1 with little difference on $K^{\prime}_{+2}$ *k*′_+2_ (Table 1). The slow phase measured here has been seen previously for non-muscle and slow striated muscle myosin isoforms [26, 30]. The relative amplitude of the slow phase (~10% of the total amplitude for both α- and β-S1) defines the fraction of the actin·S1 complex that has a closed nucleotide pocket (equilibrium constant *K*_α_, Scheme 1) that must isomerize (with rate constant *k*_+α_) before ATP can bind [26]. The closed-pocket form of actin·S1 is not illustrated in Fig. 1 because it is not on the primary cross-bridge cycle.

### ADP release from actin·S1 is >ten-fold faster for α- than β-S1

**Fig. 4:**
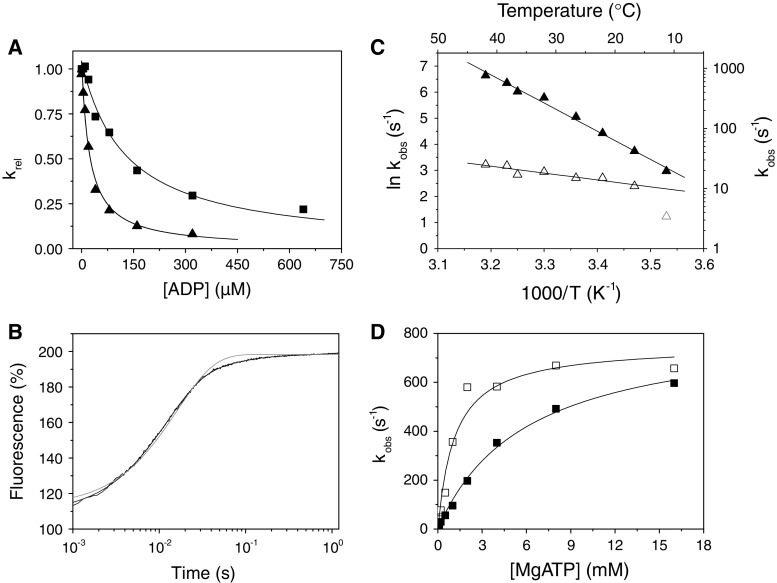
ATP-induced dissociation of pyrene-actin·S1 (α or β) in the presence of ADP. **a** 0.1 μM pyrene-labeled actin·S1 was rapidly mixed with 25 μM ATP (α) or 50 μM ATP (β) and variable ADP (20°C). The data were fitted to a single exponential, and *k*_rel_ (= *k*_obs_/*k*_0_ where *k*_0_ = *k*_obs_ with [ADP] = 0) was plotted against [ADP]. Fitting the data to *k*_rel_ = 1/(1 + [ADP]/$k_{5}^{\prime}$), resulting in an apparent affinity ($k_{5}^{\prime}$    = 127 ± 16 μM for α-S1 (*filled square*) and 23 ± 3 μM for β-S1 (*filled triangle*). Table 1 shows the average value of *K*′_5_ (*n* = 3). **b** 0.5 μM actin·β-S1 was pre-incubated with 100 μM ADP and then rapidly mixed with a large excess of ATP (4 mM ATP). The resulting pyrene fluorescence transient was best described by a two exponential fit (*solid line*). For comparison, a single exponential fit is also shown (*grey line*). The fast component with *k*_obs_ = 84 s^−1^ (amp +38%) defined the rate constant of ADP release ($K^{\prime}_{5}$) whereas the slower phase defines the rate constant of nucleotide pocket opening to allow ADP release *k*_+αD_ 15.9 s^−1^ (amp +6%). **c** Temperature dependence of ATP induced dissociation of actin·β-S1 in the presence of ADP as in Fig. 4b with *k*_obs_ (fast (*filled triangle*) $k_{+5}^{\prime}$   and slow (*open triangle*) *k*_+αD_ phase) as a function of temperature. From the slope the activation energy can be calculated. *E*_a_ = 89.8 and 38 kJmol^−1^ for the fast and the slow phase, respectively. **d** ATP-induced dissociation of actin·α-S1 in the presence (*filled square*) or absence (*open square*) of 125 μM ADP at 12°C. A hyperbolic fit gives at high [ATP] similar maximum rate constants for the dissociation of actin·α-S1 in the presence or absence of ADP ($k_{+2}^{\prime}$  = 754 and 837 s^−1^ respectively), indicating that ADP does not limit the maximum rate constant for α-S1

We measured the ADP-affinity ($K^{\prime}_{5}$) and ADP release rate constant (*k*′_+5_) for the two cardiac isoforms in order to establish whether ADP release could be rate-limiting for the cross-bridge detachment and thus the maximum shortening velocity of cardiac muscle fibers. The equilibrium constant for ADP binding to α and β actin·S1 can be estimated by measuring the ADP inhibition of the observed rate constant of ATP-induced dissociation of actin·S1 as described above. The results are depicted in Fig. 4a and typical transients are shown in supplementary Fig. S1A. Figure 4a shows that the *k*_obs_ of the fast phase of the ATP induced dissociation reaction decreased as the ADP concentration increased. The data in Fig. 4a clearly show that actin·β-S1 binds ADP much more tightly than actin·α-S1. Analysis of the data shows that *K*′_5_ = 127 μM for α-S1 and 23 μM for β-S1. This tight affinity for ADP is similar to that reported for β-S1 isolated from slow muscle [26] but α-S1 behaves more like fast skeletal muscle myosin which typically has a much lower affinity for ADP (see mean values in Table 1) [10].

Actin·β-S1 has a tight affinity for ADP and therefore a stable actin·S1·ADP complex can be formed in vitro for β-S1. Hence, saturating actin·β-S1 with ADP and then competing ADP off with a large excess of ATP allows the rate constants for ADP release to be measured. 0.5 μM actin·β-S1 was pre-incubated with 100 μM ADP and then rapidly mixed with a large excess of ATP (4 mM ATP). The resulting pyrene fluorescence transient can be described by a two exponential fit (Fig. 4b shows a one and a two exponential fit of the data). The fast component with *k*_obs_ = 84 s^−1^ (amp +38%) defines the rate constant of ADP release ($K^{\prime}_{5}$) whereas the slower phase defines the rate constant of nucleotide pocket opening to allow ADP release *k*_+αD_ 15.9 s^−1^ (amp +6%). For β-S1, the rate constant for ADP release (mean value $K^{\prime}_{5}$  = 92 ± 8 s^−1^) is very similar to ADP release from actin·β-S1 isolated from bovine masseter or bovine cardiac muscle [26, 28] as is the rate constant of nucleotide pocket opening (*k*_+αD_ = 15 ± 1 s^−1^). The relative amplitude of the two phases defines the fraction of the actin·S1·ADP complex in the open and closed pocket forms (Scheme 1); thus the equilibrium constant, *K*_αD_ is 0.38/0.06 = 6.3 and a mean value of 7 ± 1 was observed over a series of three preparations. The temperature dependence of the observed fast and slow rates (Fig. 4c) allows one to determine the activation energy values *E*_a_ = 89.8 kJ (fast phase) and 38 kJ (slow phase). The ratio of amplitudes of the two phases, *A*_fast_:*A*_slow_ had a small dependence on the temperature with *A*_f_:*A*_s_ = 6.4 at 15°C, reducing to 3.5 at 40°C. At 37°C the rate constant of ADP release, $K^{\prime}_{5}$  = 575 s^−1^ and the isomerization rate constant *k*_+αD_ = 25 s^−1^. The same measurement cannot be made for α-S1 as the affinity of S1 for ADP and actin in the ternary actin·α-S1·ADP complex is too weak at accessible protein concentrations (see below).

The low affinity of ADP for actin·α-S1 predicts that the ADP release rate ($K^{\prime}_{5}$) for α-S1 will be faster than the maximal rate of ATP induced actin·S1 dissociation ($K^{\prime}_{+2}$) and therefore too fast to be measured by ADP displacement with excess ATP. This was shown to be true in Fig. 4d where the experiment of Fig. 3b was repeated in the presence of 125 μM ADP (see supplementary Fig. S1B for sample transients). Here we used a lower temperature (12°C) to improve the precision of the maximum value of *k*_obs_ that can be measured. At 20°C the maximum *k*_obs_ (1,500 s^−1^) is near the limit of the resolution of the instrumentation. A plot of the *k*_obs_ versus [ATP] is shown in Fig. 4d and the maximum value for *k*_obs_ saturates at approximately the same value in the presence and absence of ADP (754 ± 50 and 837 ± 84 s^−1^). Thus ADP release does not limit the maximum *k*_obs_ for ATP binding to actin·α-S1·ADP. ADP binding is therefore a rapid equilibrium step and $K^{\prime}_{5}$ is much greater than $K^{\prime}_{+2}$ (>750 s^−1^ at 12°C or >1,500 s^−1^ at 20°C). As noted above, this behavior of α-S1 is similar to that of fast skeletal muscle myosin [10] and not of any previously documented slow or cardiac isoform. The two cardiac myosin isoforms are therefore predicted to have very different lifetimes of the force-holding states (actin·S1·ADP and actin·S1) and these differences predict a faster shortening velocity for muscle fibers expressing α-MyHC than those expressing only β-MyHC; consistent with measurements of cardiac shortening velocity [31].

### β-S1 binds actin five- to ten-fold tighter than α-S1

**Fig. 5:**
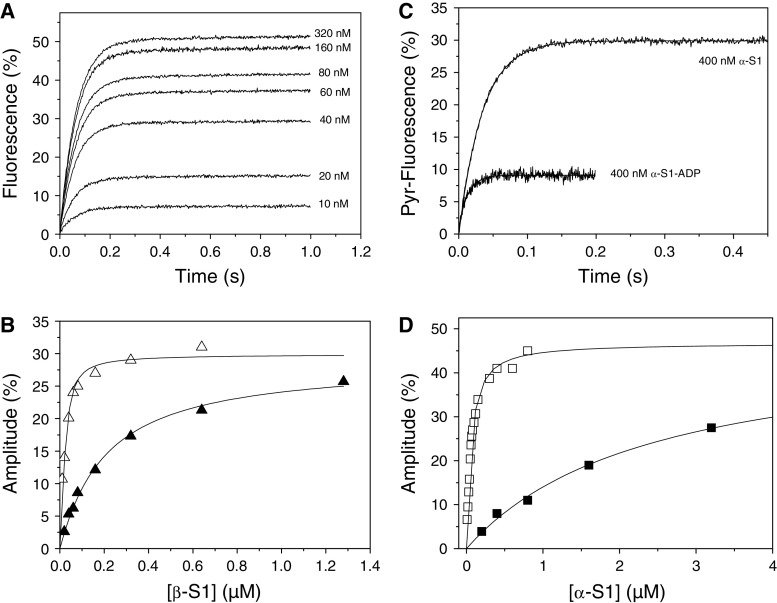
Titration of actin with cardiac myosin isoforms. **a** Fluorescence transients observed when 20 μM ATP was used to dissociate 30 nM actin from increasing concentrations of β-S1. The fluorescence was fitted to a single exponential, the *k*_obs_ (= 18 s^−1^) was constant and the amplitude increased with increasing [S1]. **b** A plot of the amplitudes in *A* versus [β-S1] (*open triangle*) and similar data for β-S1 in the presence of 500 μM ADP (*filled triangle*). Note that plotted concentrations are before mixing. The result was fitted to the quadratic equation describing the binding isotherm (see “Experimental” section) resulted in a *K*_A_ = 8 nM and *K*_DA_ = 190 nM for β-S1. **c** Example traces used for the results in (**d**): 30 nM actin was incubated with 400 nM α-S1 or 400 nM α-S1·ADP before rapidly mixing with 20 μM ATP or 250 μM ATP. Without ADP the fluorescence transient was fitted to a single exponential with *k*_obs_ = 29 s^−1^ and Amp = 30%. In the presence of ADP the fluorescence transient, fitted to a single exponential, resulted in *k*_obs_ = 66 s^−1^ and Amp = 11%. The large difference in measured fluorescence amplitude is due to the weak affinity of α-S1-ADP for actin. **d** A similar plot as B for α-S1 (*open square*) and α-${\text{S}}1 \cdot {\text{ADP}}$ (*filled square*) in which ADP was 1 mM resulting in *K*_A_ = 44 nM and *K*_DA_ = 2.4 μM. Plotted concentrations are before mixing. Table 1 gives the average values of 2–3 independent measurements of *K*_A_ and *K*_DA_

The affinity of actin for S1 in the rigor, actin·S1 complex (*K*_A_), and in the actin·S1·ADP complex (*K*_DA_) gives an indication of the strength of the actin–myosin bond that holds the force that myosin generates. It also yields the value for the thermodynamic coupling between actin and nucleotide binding (*K*_AD_/*K*_D_ = the extent to which actin decreases the affinity of S1 for ADP and conversely *K*_DA_/*K*_A_ = the extent to which ADP decreases the affinity of S1 for actin); a key characteristic of the mechanical activity of a myosin isoform [30]. The thermodynamic coupling (*K*_AD_/*K*_D_) between actin and ADP binding to S1 can be validated by measuring the affinity of S1 for actin in the presence and absence of ADP (*K*_DA_/*K*_A_). Phalloidin-stabilized pyrene-actin (15 nM) incubated with various concentrations of α-S1 or β-S1 was mixed with ATP and the amplitude of the dissociation reaction was used to estimate the fraction of actin bound to S1. Figure 5a shows a series of transients observed on ATP induced dissociation of β-S1 from pyrene-labeled actin·β-S1 at a fixed 30 nM pyrene-actin with increasing S1 concentration. Since the ATP concentration was fixed, the *k*_obs_ values did not change but the amplitude increased as the S1 concentration increased until the actin was saturated. A plot of observed amplitude versus [S1] (given in Fig. 5b) shows that β-S1 has a tight actin binding (*K*_A_ = 8 nM). When the experiment was repeated in the presence of saturating ADP the affinity was reduced ~20-fold (*K*_DA_ = 190 nM). The same measurement was repeated for α-S1 and Fig. 5c shows a transient observed in the presence and absence of 1 mM ADP. This shows a much smaller amplitude in the presence of ADP. The plot of amplitude of the observed transient versus [α-S1] shown in Fig. 5d gives the affinities of α-S1 for actin as *K*_A_ = 44 nM and *K*_da_ = 2.4 μM.

The average actin-affinity measured for β-S1 (8 ± 2 nM) is similar to the affinity of bovine β-S1 (7 nM) whereas the affinity for α-S1 (37 ± 11 nM) is similar to rabbit fast muscle S1 (33 nM) (Table 1) [26]. In the presence of saturating ADP concentrations affinities were reduced 50-fold for α-S1 (1.84 μM) and 24-fold for β-S1 (Table 1). The weak affinity of S1 for actin is again a feature common to α-S1 and fast muscle myosin isoforms and distinct from slow muscle-derived β-S1.

### ATP hydrolysis is ten-fold faster for α-S1 than β-S1

The isometric force that a muscle can generate is proportional to the number of attached myosin cross-bridges in the sarcomere. While ADP release and ATP binding limit the exit from the attached states, entry into the attached states is influenced by other steps in the cross-bridge cycle, such as myosin access to actin-binding sites (at the high local concentration of actin in the sarcomere this is largely limited by the geometry of the thick and thin filaments and sliding velocity [32]), the release of P_i_ that commits myosin to strongly bind actin, and the hydrolysis step which defines the time of the myosin recovery stroke (Step 3) before it can again bind to actin [32].

**Fig. 6:**
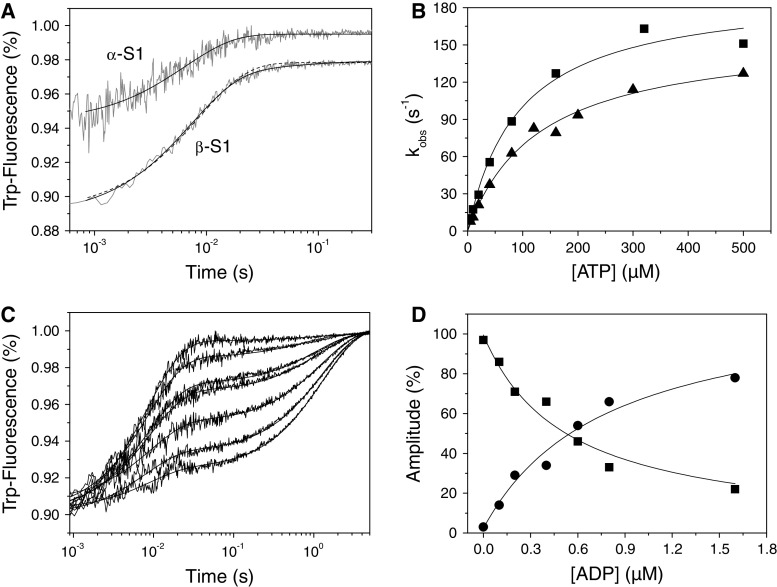
Binding of ATP or ADP to cardiac S1. **a** Tryptophan fluorescence traces observed upon rapidly mixing 0.2 μM α- or β-S1 with 500 μM ATP. For α-S1 the fluorescence traces were best fit by a single exponential, *k*_obs_ = 151 s^−1^ (amp = 5.1%), whereas for β-S1 the fluorescence traces (offset by −0.02) were best fit by a double exponential (*solid line*), *k*_obs_ = 124 s^−1^ (amp = 8.4%) and 19 s^−1^ (amp = 0.8%). Note that a single exponential fit (*dashed line* *k*_obs_ = 117 s^−1^) is also shown for comparison. **b** The dependence of *k*_obs_ on [ATP] yields *K*_1_ *k*_+2_ = 2.7 μM^−1^ s^−1^ for α-S1 (*filled square*) and *K*_1_ *k*_+2_ = 1.23 μM^−1^ s^−1^ for the fast phase of β-S1 (*filled triangle*). At high ATP-concentrations *k*_obs_ saturates at 196 s^−1^ (α-S1) and 158 s^−1^ (β-S1). The slow phase measured for β-S1 saturates at ~26 s^−1^. **c** Tryptophan fluorescence traces observed after incubating 0.2 μM β-S1 with variable [ADP] (0–1.6 μM) before rapidly mixing with 100 μM ATP. The data fit best to a sum of two exponentials with *k*_obs_ = 112 s^−1^ (fast phase) and 0.8 s^−1^ (slow phase). **d** Dependence of the relative amplitudes of the two exponentials measured in Fig. 6c on ADP concentration (before mixing). The data are fitted to Eqs. 5A and 5B (“Experimental” section) with a *K*_5_ = 0.53 μM (fast phase, *filled square*) and 0.8 μM (slow phase, *filled circle*)

Myosin S1 contains tryptophan residues that have intrinsic fluorescence and can be used to detect both the ATP-binding and the ATP hydrolysis steps (Fig. 1 Steps 1–3) in the absence of actin. Upon mixing 500 μM ATP with 0.2 μM S1 the tryptophan fluorescence signal of showed a single phase for α-S1, whereas for β-S1 the fluorescence signal showed two phases, a fast phase similar to α-S1 and an additional slow phase (Fig. 6a). The fast phase had a *k*_obs_ of 151 s^−1^ (amp 5.1%) for α-S1 and 124 s^−1^ (amp 8.4%) for β-S1. The fast phases were hyperbolically dependent upon ATP concentration as shown in Fig. 6b and the data were fitted to Eq. 3, as defined in the legend.

The slow phase observed for β-S1 was small and not always easy to observe (see single exponential fit in Fig. 6a) but was consistently observed when the S1 was treated with apyrase to remove any contaminate ADP. A similar slow phase was previously seen in other β-S1 preparations from Bovine masseter and rabbit soleus muscles [26, 29]. It was also clearly present in β-sS1 preparation (see supplementary Fig. 2E). The slow phase was independent of ATP concentration above 100 μM with *k*_obs_ = 19 s^−1^ and an amplitude of 0.8%.

For β-S1 derived from slow skeletal muscle a similar slow phase of the fluorescence change has been assigned to the hydrolysis step (Step 3; *k*_obs_ = *k*_+3_ + *k*_−3_) [26]. To ascertain if this was also true for human β-S1 we used quenched-flow methods to directly measure the rapid burst of ATP hydrolysis. The mouse C_2_C_12_ cell expressed protein is limited in both quantities and the S1 concentrations that can be achieved. We therefore could not complete a full quenched-flow study but simply attempted to distinguish between the hydrolysis step coinciding with either the fast or slow phase of the fluorescence transient. The maximum β-S1 available after mixing was 3.5 μM, and this was mixed with a ten-fold excess of ATP and incubated at 20°C before quenching the reaction in acid and analyzing the amount of ADP generated. A rapid initial burst of ADP production was observed and at 350 ms with a burst size of 0.59 ADP/S1. Three time points were chosen at 16, 33, and 68 ms to give the best chance of distinguishing the two possibilities. The results gave the ADP produced as 16, 43, and 61% of the burst amplitude. A single exponential fit to the data suggests a *k*_obs_ value of ~15 s^−1^. Using the values given in Table 1 the expected *k*_obs_ for the fast phase is *K*_1_ *k*_+2_[ATP] = 1.5 × 10^6^ × 35 × 10^−6^ = 52.5 s^−1^. This predicts the ADP generated at the three time points of 57, 82 and 95%. The ADP burst rate is thus a third that of the predicted fast phase fluorescence. The quality and quantity of the data does not warrant a detailed fitting of the data to a model. However a simple M → M·ATP → M·ADP·P_i_ model where the first step is the irreversible ATP binding at *K*_1_ *k*_+2_[ATP] = 52.5 s^−1^ and the second step is the maximum observed value of *k*_obs_ for the slow phase of 17 s^−1^ (Table 1) predicts a burst with a half time of ~66 ms and thus a *k*_obs_ of 10.5 s^−1^. A much closer fit to the 15 s^−1^ observed than the predicted fast phase fluorescence. The data are therefore compatible with the hydrolysis step being slower than the fast fluorescence transient (assigned to ATP binding). The similarity of the data presented here together with that reported for tissue-purified β-S1 suggests that the hydrolysis step does correspond to the slow fluorescence change but given the error on each measurement the assignment is provisional.

Quenched-flow measurements with α-S1 at a single time point of 13 ms indicated 33% of the burst amplitude, which is much higher than observed for the β-S1. This is compatible with a *k*_obs_ of 85 s^−1^ and a predicted value for the fast phase of 100 s^−1^. There was no observable slow phase fluorescence for the α-S1 and therefore we assume the maximum observed rate constant for the fast fluorescence transient corresponds to the hydrolysis step. Further evidence for this assignment of the single phase for α-S1 is that the amplitude of the fluorescence change decreased from 10% at low ATP (<100 μM) to ~6% at higher ATP (>300 μM). This could indicate the presence of a fast fluorescence change associated with ATP binding which becomes lost in the dead time of the system at high ATP concentrations. For α-S1, these characteristics are again similar to the characteristics of fast skeletal muscle myosin [33, 34]. Thus α- and β-S1 differ greatly in the speed with which the myosin can complete the hydrolysis step/recovery stroke before it can once again bind to actin.

There is only a very small change in tryptophan fluorescence when ADP binds to cardiac S1. Therefore, the larger fluorescence change which occurs on ATP binding can be used to measure the displacement of ADP from S1. Therefore, in order to measure the ADP affinity (*K*_5_) and ADP off-rate constant (*k*_+5_), ADP was displaced by addition of a large excess of ATP [26]. Figure 6c shows the fluorescence transients observed on displacing ADP (0–1.6 μM) from 0.2 μM β-S1. The transients show a fast and a slow phase; the fast phases represent ATP-binding to free S1 and the amplitude decreases as the ADP concentration increases. The slow phase is the displacement of ADP from S1·ADP by ATP and the amplitude increases with increasing ADP concentration. The ratio of the two amplitudes therefore represents [S1]_free_/[S1·ADP]. Fitting the amplitude dependence on [ADP] to Eq. 5A and 5B (Fig. 6d) gives an estimate of the ADP-affinity *K*_5_ = 0.5–0.8 μM for β-S1. The *k*_obs_ of the slow phase was independent of ADP concentration and ATP >100 μM and therefore represents the ADP off-rate constant *k*_+5_ = 0.8 s^−1^. The ADP affinity was weaker for α-S1 (*K*_5_ = 2.8 μM) with a faster ADP release rate *k*_+5_ = 2.7 s^−1^.

### The humanized ELC form, β-sS1, is kinetically similar to β-S1

**Fig. 7:**
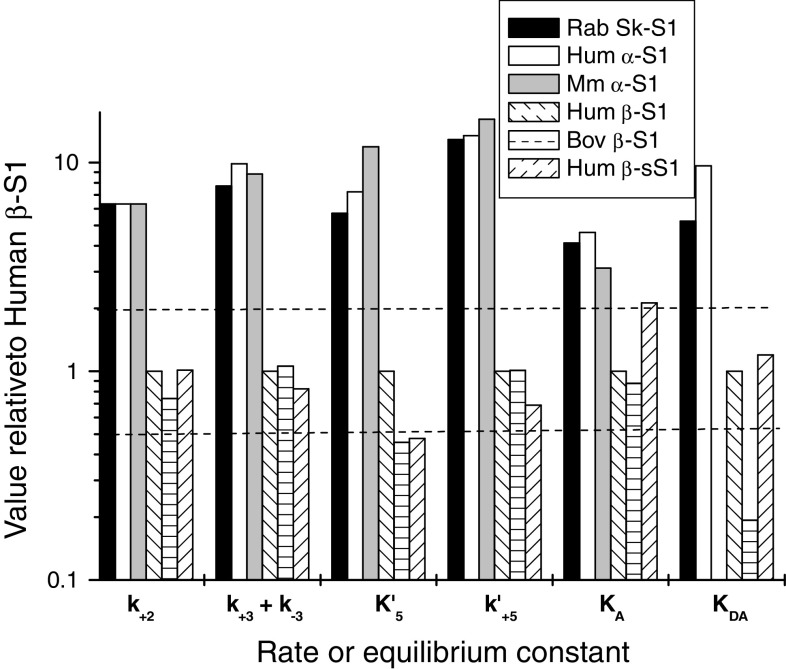
Comparison of the rate and equilibrium constants for myosin S1 relative to those of Human β-S1. The values listed in Table 1 which discriminate between the α and β isoforms were divided by the equivalent value for human β-S1 and plotted on a log scale. *Values* contained between the two *horizontal lines* are within a factor of 2 of the values for human β-S1. This region contains almost all of the human and bovine β-S1 and bovine β-S1 isoform data and excludes all of the α-S1 and rabbit skeletal S1 data. All values plotted for the human and mouse α isoforms lie outside this range and are at least three-fold larger than the value for β-S1. Values for Rabbit skeletal S1 are shown for comparison and are similar to the α isoform values in each case. Other values in Table 1 are within a factor of 2 of the human β-S1 values for all isoforms

We repeated all of the measurements described above for the “humanized” β-sS1 construct. In almost all cases, the data show little difference between the two β-constructs (see example data in Supplementary Fig. S2A–F summarized in Table 1 and Fig. 7). The constants, *K*_1_ *k*_+2_ and *k*_+2_ for S1 alone do not differ between the two constructs. All other constants differ by no more than two-fold with the exception of *k*_+αD_ which is seven-fold faster for β-S1 than for β-sS1. Thus, as seen for other myosin constructs, in the absence of load the light chains have little influence on the biochemical kinetic properties of S1.

### Mouse cardiac α-S1 is similar to the recombinant human α-S1

In order to compare recombinant protein with myosin purified from cardiac tissue, we isolated myosin from mouse heart, which almost exclusively expresses the α myosin isoform [35]. Myosin was isolated from a single mouse heart and was digested with chymotrypsin as described above. The S1 thus generated was active for only a few days, therefore the preparation from heart to S1 was completed in 24 h and used without extensive purification. The digested myosin was simply spun down to remove insoluble material. All measurements were completed within 48 h of digestion of the myosin. Figure 2c shows a Coomassie-stained gel of the purified α-myosin and α-S1. Almost all of the measurements reported above were repeated on mouse cardiac α-S1. Examples of data are shown in Supplementary Fig. S3 and the data are summarized in Table 1 and Fig. 7. These show that the properties of tissue-purified mouse α-S1 and the human α-S1 differ by no more than two-fold for any of the parameters measured, with the exception of 1/$K^{\prime}_{1}$. However, mouse α-S1 is quite distinct from β-S1.

## Discussion

We present here the first detailed kinetic characterization of human cardiac myosin isoforms in order to begin to understand the role of the different isoforms in contraction and structure/function relationships of the striated myosin II isoforms. Detailed kinetic studies of β-S1 from the heart or slow skeletal muscle of the rabbit, rat, cow and pig have been reported [26, 28, 29], but there have been no published kinetic studies of human β-S1 or any α-S1.

As shown in Table 1 the kinetic properties of the recombinant human β-S1 cardiac motor appear to be very similar to the other β-type myosins isolated to date [26]. These are characterized by a tight affinity of the actin·β-S1s for ADP ($K^{\prime}_{5}$), a slow rate constant of ADP release ($K^{\prime}_{+5}$), a slow rate constant of the ATP hydrolysis step (*k*_+3_ + *k*_−3_) and a tight affinity of the β-S1s for actin in the presence and absence of ADP (*K*_DA_ and *K*_A_, respectively). These properties appear therefore to be associated with the β-MyHC and not with the species, the method of generation or the associated light chains. In contrast, the α-S1 differs from the β-S1 in the same set of parameters with a 7- to 15-fold weaker affinity for ADP ($K^{\prime}_{5}$) due to a very fast ADP release from actin·α-S1 ($K^{\prime}_{+5}$), a ~ten-fold faster ATP hydrolysis step (*k*_+3_ + *k*_−3_) and five- to ten-fold weaker affinity for actin (*K*_A_ and *K*_DA_). These properties are shared by the both the recombinant human cardiac and mouse α-cardiac isoform (Table 1), confirming that these properties are a feature of the α isoform and not the method of preparation. In each of these parameters, the α-S1 is far more similar to a fast skeletal muscle isoform such as the well-defined rabbit IIb isoform listed in Table 1. This is surprising, as the human α and β motor domains share 91% sequence identity whereas both are only 80% identical to the human fast muscle isoforms (IIa, IIb and IIx/d; [4]).

The kinetic differences outlined above distinguish the α and β isoforms, since all other parameters listed in Table 1 are similar (within a factor of 2) for the two isoforms. This is illustrated in Fig. 7 where the parameters that differ by more than two-fold between α and β-S1 are plotted along with values from rabbit fast muscle S1, mouse α-S1 from heart and bovine β-S1 from masseter muscle. This plot shows how these parameters are similar for the two α isoforms from mouse and human and the faster skeletal S1 from rabbit, yet distinct from bovine β-S1 and the human β-S1 and sS1. This grouping of kinetic properties allows an analysis of the common features of α and β motor domain sequences to define which residues may be responsible for the change in the affinity of S1 for actin, the affinity of actin·S1 for ADP or the ATP hydrolysis step. A sequence alignment of the α- and β-isoforms from the mouse, rat, and human along with the bovine β isoform (Fig. S4) results in a consensus sequence for the α and β isoforms which reduces the difference in α and β from 80 residues between the two human isoforms to 40 conserved differences in the motor domain among the groups (plus 7 in the first IQ domain). Forty differences between α and β are too many to attempt a systematic site-directed mutagenesis approach to define the differences. However, the sequence differences are not uniformly scattered through the motor domain. For example, there are no differences in the RLC-binding domain and none in the converter region. The well-known variable surface loops contain differences (five in Loop 1 and ten in Loop 2) and are known to modulate the properties of many myosins [36, 37]. Loop 1 can modulate the affinity of actin–myosin for ADP [38, 39] and Loop 2 the affinity of myosin for actin [40, 41]. However, studies of mouse cardiac muscle isoforms in which the Loop 1 and 2 chimeras were created showed few changes in behavior arguing that we should look elsewhere for the significant changes in the structure of the α and β isoforms [42].

There are no sequence changes in residues in direct contact with nucleotide or Mg^2+^ and therefore no simple explanation for the changes in nucleotide affinity. The 50-kDa domains (between Loop 1 and Loop 2) show the largest number of differences between the α and β isoforms (16 differences). These are highlighted in Supplementary Fig. S5. Of these, four residues appear to be in sites that could make direct contact with actin; Q595E (using the β myosin residue numbers with the β residue quoted first), and the cluster A423S, X421Y and N416S (where X is a variable I/V/A or S in the β myosins). These sites are in a position to directly influence the S1 affinity for actin but without a high-resolution structure of the actin–myosin interface further comment is only speculation.

A feature of α (and fast muscle) isoforms is that the ability of actin to displace ADP and vice versa (the ADP and actin coupling $K^{\prime}_{5}$/*K*_5_, *K*_DA_/*K*_A_, [30]) is stronger than in the β isoforms suggesting that the communication between the two sites is altered. This communication goes through the 50-kDa domain and one area of interest is in the region 297–327. This corresponds to exon 7 of *Drosophila* myosin II and is a region that is alternately spliced to generate different *Drosophila* isoforms and can contribute to the coupling of ADP and actin affinity to S1 [43]. Residue changes in this region between cardiac β versus α, respectively are I303V, I313V, and T318V and T319S. The T318V change is the only non-conservative change here. This sequence region also has human cardiomyopathy mutations F312C and V320M in cardiac β-myosin, adjacent to the residues which differ with respect to α myosin.

Exon 7 ends in the middle of helix K and a short loop joins this to helix L. There is a variable cluster of three residues in helix L, 347–349 that is NSM/I in β and AGV in α. This triplet is variable across all myosins and lies between two highly conserved lysines at 346 and 351. K351 in β myosin is a human cardiomyopathy site. We have argued previously [43] that this exon 7 region and its links to helix L were important in coupling the nucleotide-binding pocket and the actin-binding sites and therefore the same argument can be made for the role of these sequence changes in cardiac myosin.

There is an interesting cluster of three amino acids that differ between cardiac α and β isoforms after Loop 2. The helix that follows Loop 2 ends at residue 664 and this has a conservative T (in β) to S (in α) change, this is followed by strand 3 of the central beta-sheet and then a short linker and the SH-2 helix. The linker has a highly conserved PNEXKXPG sequence where Xs represent the β to α isoform changes T678R and S680A. The conserved E677 makes a salt bridge to Switch 1 (238) and this salt bridge is present in all crystal structures so far examined. The conserved K679 makes a hydrogen bond to the backbone of Switch 1. This link to Switch 1 is intriguing and it is possible that the two mutations can modulate the salt bridge and thereby alter Switch 1 movement and hence Switch 2. Switch 1 is a key part of the nucleotide-binding site, therefore changes here could alter either ADP release, the hydrolysis step or both. Counter to this argument is the observation that the R678 and A680 α isoform sequence is not conserved in fast muscle myosins. For example the R678 and A680 residues are both replaced in mouse and human MyHC IIa isoform by threonine. This is more similar to the β cardiac myosin sequence (T and S) yet the fast skeletal isoforms share the kinetic properties of α cardiac myosin.

The overall contractile character α-S1 appears to be like that of a fast rabbit skeletal muscle isoform (MyHC IIx). This comparison also holds true for other recombinant human fast skeletal myosin motors prepared in the same way (Bloemink et al., in preparation). However, actin·α-S1 does share the presence of a slow phase in nucleotide binding ($K^{\prime}_{\alpha 1}$) with actin·β-S1. This slow phase is attributed to a fraction of the actin·S1 with a closed nucleotide pocket that must open ($K^{\prime}_{\alpha 1}$) before ATP can bind or ADP can be released. This has only been observed in slow muscle and non-muscle myosin isoforms and is hypothesized to be related to a 2-step load dependent ADP release mechanism [26]. For the β-S1, we observed two well-defined steps in ADP release (Step 5′ and αD) with an equilibrium constant $K^{\prime}_{\alpha {\rm D}}$ ≈ 5. The single fast step seen for ADP release from actin·α-S1 is typical of a fast muscle isoform; it is believed that for fast muscle isoforms an isomerization step is present but the equilibrium constant is >10, thus the complex cannot be formed by simply adding ADP to the rigor complex [44]. By analogy, the same argument may hold for the cardiac α-S1.

### Implications for cardiac muscle fibers

Differences in transient kinetic properties between the α and β isoforms are more striking than might have been expected from the two- to three-fold differences in shortening velocity, in vitro motility and ATPase activity reported in the literature for the α and β isoforms from other species [5, 12, 35, 45–48]. Single molecule mechanical studies of tissue isolated α- and β-myosins have shown that the step size and unitary force do not change. However, the lifetimes of the attached states do differ two-fold [6]. Using a higher time resolution laser trap Capitanio et al. [49] reported that the actin–myosin mechanical event can be resolved into two components, the first limited by ADP release and the second limited by the concentration of ATP used. For full-length β-myosin isolated from rat soleus muscle, the ADP limited step was ~42 s^−1^ compared to the 400 s^−1^ for a fast type myosin. For slow muscle myosins (the β isoform) the ADP release step measured in solution ($K^{\prime}_{+5}$) correlates with the event that limits the maximum shortening velocity [26, 29]. For fast muscle myosins, the 400 s^−1^ ADP-linked event in the laser trap is slower than the value of $K^{\prime}_{\alpha {+5}}$ observed in solution for ADP release. It is therefore thought to be an isomerization that is not accessible by simply adding ADP to actin·S1 and can only be accessed following ATP hydrolysis and P_i_ release. In several non-muscle myosins the ADP release event is load dependent and slows down by 5- to 100-fold [50, 51] when the myosin is bearing a load equivalent to isometric force. This marked difference in the ADP release event between fast and slow muscle myosins is compatible with our β-S1 data which shows a >ten-fold slower ADP release for β-S1 than either α or that reported for fast skeletal muscle myosins [10, 26]. The ATP limited steps observed in the optical trap are more similar for fast and slow muscle myosins as we also observed for α- and β-S1. No fast laser trap measurements have been made for any α-myosin.

Like the single molecule measurements and our solution data, mechanical measurements on human cardiac myofibrils and cardiomyocytes show that the differences between the myosins may be larger than expected from ATPase and motility data alone. Studies of single human atrial (predominantly α-myosin) and ventricular (predominantly β-myosin) myofibrils show that while the isometric tension varies by 10–20% (108–125 mN mm^−2^) the rate of tension development after a period of rapid shortening (known as the rate constant *k*_tr_) is five-fold faster for atrial than for ventricle samples [52]. Similarly a study of the myosin isoform content of human atrial cardiomyocytes from patients with atrial fibrillation or dilation have shown that the β-myosin content increases from 10% in normal hearts to up to 60% [53]. Again, while the isometric force generation was not correlated with β-myosin content, the rate of force development (*k*_tr_) decreased from 10 s^−1^ for cardiomyocytes with 10% β-myosin to 1 s^−1^ for 60% β-myosin [53]. A direct correlation of *k*_tr_ with solution data is not currently possible but Piroddi et al. [52] did establish that the differences in *k*_tr_ were not attributable to changes in calcium binding or release from the thin filaments. Currently *k*_tr_ is thought to be limited by cycling cross-bridges as they change from unloaded to loaded isometric conditions. This slowing down of loaded contractions is believed to be linked to load-limited ADP release.

As discussed above, the rate of ADP release from actin·S1 does appear to be the event that limits the shortening velocity in slow muscle and our low value of the ADP release in β-S1 is consistent with this view. For fast muscle myosin ADP release as measured in solution is very fast and too fast to limit the shortening velocity. Our results with α-S1 are consistent with this view. The data in Fig. 4c show the value of the ADP release rate constant ($K^{\prime}_{+5}$) over the temperature range from 10 to 40°C. The value of $K^{\prime}_{+5}$ increases almost seven-fold between 20 and 37°C (from 84 to 575 s^−1^) and therefore predicts a similar increase in shortening velocity over this temperature range.

Using the rate constants in Table 1 extrapolated to physiological ATP concentration (5 mM) and assuming unlimited actin concentration (the situation in a rapidly contracting muscle fiber), we can estimate the time that the two isoforms spend strongly bound to actin $(1/K^{\prime}_{{ + 5}}  + 1/(K^{\prime}_{1} K^{\prime}_{{ + 2}} [{\rm{ATP}}])/(1 + K^{\prime}_{1} [{\rm{ATP]))}}$ and the time free from actin (1/(*k*_+3_ + *k*_−3_)) at 20°C. By these calculations α-S1 spends ~0.7 ms strongly bound to actin and ~6.0 ms unbound while β-S1 spends ~12.9 ms strongly bound and ~67.0 ms unbound. In this modeled contractile cycle the two motors have a >ten-fold difference in cycle time: 6.7 (0.7 + 6.0) ms for α-S1 and 79.9 (67 + 12.9) ms for β-S1. Thus, although α and β spend very different lengths of time in these bound and unbound states they have a similar duty ratio (bound/bound + unbound) which remains low at ~10% for α-S1 and ~16% for β-S1. This is compatible with observations that the unitary force per myosin does not change and that the isometric force is not greatly altered. Thus, the number of myosin heads attached is similar and the force per head is also the same. The consequences of a similar duty ratio but a longer lifetime of the attached force-holding states has implications for the rate of activation and relaxation of the thin filament. If strongly bound myosin heads are a significant component of the thin filament activation process then actin filaments with an attached β-myosin will be much slower to relax than with an α-myosin. Similarly, the faster cycling α-myosin could activate the thin filament faster than β-myosin with an equivalent level of calcium present. These cooperative effects are unlikely to be linearly related to the fraction of α and β present in a given tissue. In several mechanical assays, the presence of a small amount of one isoform has a dominant effect on the overall phenotype observed and this is particularly true of power output and the rate of relaxation [18].

The roles of cardiac myosin isoforms have been explored in cell and transgenic studies. For example, persistent expression of α-MyHC in hearts of rabbits (which normally express β-MyHC) that have tachycardia-induced failure or myocardial infarction provides cardioprotection [54, 55]. Recently, gene transfer of human α- and β-MyHCs into adult cardiac myocytes was performed, achieving the transfer of human α into failing human and rabbit cardiac myocytes and human β into rat cardiac myocytes. The investigators found that human α-MyHC representing 21% of total MyHC 24 h after transduction or 30% of total after 48 h increased the degree of contractility as well as the kinetics of contraction and relaxation without affecting Ca^2+^ transients [56]. Transfer of human β-MyHC into the predominantly α-MyHC rat myocytes where human β-MyHC represented 18 or 38% of the total MyHC decreased the amount of contraction by 42 and 57%, respectively [57]. These effects were also Ca^2+^ independent. Together, these results support the notion that the myosin composition of the human heart influences cardiac function. Understanding how the two myosins work together will be an important next step.

The transient assays used here have the great advantage that they are not sensitive to the presence of contaminant myosin activity, but this makes them unsuitable for examining the potential dominant role of isoform mixture. Full-length constructs such as those described above can be used in mechanical studies these will be sensitive to the isoform mixture present and will be the subject of future work. Work with full-length human α-MyHC in adult cardiac myocytes has demonstrated dominant effects of small amounts of α-MyHC on contractility and relaxation in failing human cardiac myocytes [56]. Similarly, the study of the activation and relaxation process in solution with α- and β-myosins (and in mixtures) will be a major study that has not been addressed to date. The impact of these kinetic constants on muscle contraction lies in their contribution to defining contractile processes. These findings indicate >ten-fold differences between the cardiac isoforms α and β in the kinetic constants implicated in controlling fiber shortening velocities and cycle times, as well as subtler differences in constants influencing other contractile events. The elucidation of these values is a step towards understanding the role of myosin isoform composition in the human heart in health and disease.

### Electronic supplementary material

Below is the link to the electronic supplementary material.

Supplementary material 1 (DOC 2.12 mb)

## Abbreviations

S1: Myosin head fragment equivalent to subfragment 1
α-S1, β-S1: α or β cardiac isoforms of S1
RLC: Regulatory light chain
ELC: Essential light chain
LC: Myosin light chain
MLC1F: Alkali myosin light chain 1
MLC2F: Regulatory myosin light chain 2
MLC3F: Alkali myosin light chain 3
MLC1A: Myosin light chain 1, atrial/fetal isoform
MyHC: Myosin heavy chain
