# Supplementary material for: Identification of functional differences between recombinant human α and β cardiac myosin motors
Source: Cell Mol Life Sci. 2012 Feb 16;69(13):2261–77. doi: 10.1007/s00018-012-0927-3 (PMC3375423; doi:10.1007/s00018-012-0927-3)
Supplement: Supplementary file 1 — Supplementary material 1 (DOC 2.12 mb) [file 18_2012_927_MOESM1_ESM.doc]

Supplementary Material

**IDENTIFICATION OF FUNCTIONAL DIFFERENCES BETWEEN RECOMBINANT HUMAN α AND β CARDIAC MYOSIN MOTORS**

**John C. Deacon^1a^, Marieke J. Bloemink^1b^, Heresh Rezavandi^b^, Michael A. Geeves^2b^, & Leslie A. Leinwand^2a^**

^a^Department of Molecular, Cellular & Developmental Biology and Biofrontiers Institute, University of Colorado, Boulder, CO 80309, USA and ^b^School of Biosciences, University of Kent, Canterbury CT2 7NJ, UK

^1^ J.C.D., and M.J.B., contributed equally to this work

^2^ M.A.G and L.A.L., co-corresponding authors

**Figure S1:** ***A.*** Example pyrene fluorescence transients for data shown in Figure 4A. These were measured after rapidly mixing 50 nM pyrene-actin·β-S1with 50 µM ATP and [ADP] = 1.25, 10, 40, 80 and 160 µM. The fluorescence traces were best fitted to single exponentials from which observed rates (k_obs_) were obtained. ***B.*** Example of pyrene fluorescence traces described in Figure 4D. These were measured after rapidly mixing 50 nM pyrene-actin·α-S1 with 1 mM MgATP in the presence and absence of ADP at 12°C.

**Figure S2:** **Summary of transient kinetic data for human cardiac S1 with human light chain: β-sS1. *A.*** Example of a pyrene fluorescence transient observed for ATP-induced dissociation of 30 nM pyrene-actin·S1 by 50 µM ATP. A double exponential fitted best (solid line) and gives the k_obs_ values of 92 s^-1^ (Amp = 26%) and 18 s^-1^ (Amp = 6%). For comparison a single exponential fit (dashed line, k_obs_ = 55 s^-1^, amp = 31 %) is also shown. ***B.*** Plot of k_obs_ vs [ATP] for the fast phase (■) of the data measured in A. The k_obs_ had a hyperbolic dependence on [ATP] with a k_max_ (= k_+2_) = 1131 s^-1^ and K_0.5_ (=1/K_1_) = 0.645 mM. The slow phase (●) saturated at a k_obs_ value of 45 s^-1^. ***C.*** ADP inhibition of the dissociation of 30 nM pyrene-actin·S1 by 500 µM MgATP and increasing [ADP]. The observed transients were similar to those in *A* and a fit of the ADP dependence of the k_obs_ values (fast phase) to a hyperbola gave a half maximum inhibition (K’_5_) of 7 µM. ***D.*** Examples of fluorescence transients observed when 20 µM ATP was used to dissociate 30 nM actin from increasing concentrations of β-sS1. The fluorescence was fitted to a single exponential, the k_obs_ remained constant (18 s^-1^) and the amplitude increased with increasing [S1]. Plots of Amplitude versus [S1] (before mixing) give K_A_ (see Table 1). ***E.*** Typical tryptophan fluorescence transient for binding of 80 µM ATP to 0.1 µM S1 in the absence of actin. The transient can best be described by a double exponential (solid line) with k_obs_ values of 80 s^-1^ and 13 s^-1^ and amplitudes of 5% and 1% respectively of the final fluorescence signal. For comparison a single exponential fit is also shown (dashed line, k_obs_ = 56 s^-1^, amp = 6.5 %). ***F.*** A plot of the [ATP] dependence of the k_obs_ values, measured for the fast phase (■) in E, fitted to a hyperbolic function with k_max_ = (k_+2_) = 183 s^-1^. The slow phase (○) saturated at a k_obs_ value of ~18 s^-1^ and represents the hydrolysis rate k_+3_+k_-3_. This hydrolysis rate was verified by quench flow (k_+3_+k_-3_ = 15 s^-1^). Table 1 gives average values measured (n = 2-3).

**Figure S3: Summary of transient kinetic data for the mouse cardiac S1**

**Figure S3: Summary of transient kinetic data for mouse cardiac S1. *A.*** Typical fluorescence transients observed for ATP induced dissociation of 0.2 µM pyr-actinS1 by 25 µM and 2.5 mM ATP. Both transients are described by a two exponential fit. Fits give the k_obs_ values of 52.0 and 16.4 s^-1^ for 25 µM ATP and 380 and 52 s^-1^ for 10 mM ATP. The total fitted amplitudes were 28% with ~10 % of the total amplitude in the slow phase. The slow phase saturated at a k_obs_ value of ~50 s^-1^. Experimental conditions the same as given in the main body text but at 10°C. ***B.*** Plot of k_obs_ vs. [ATP] for the fast phase of the data in *A* at 20 and 12°C. At 20°C the k_obs_ was linearly dependent upon [ATP] (K_1_k_+2_ = 2.0 x10^6^ M^-1^s^-1^) until the reaction became too fast to measure. At 12°C the k_obs_ had a hyperbolic dependence on [ATP] with a k_max_ (= k_+2_) = 430 s^-1^ and K_0.5_ (=1/K_1_) = 0.26 mM. ***C.*** ADP inhibition of the dissociation of 0.2 µM pyrene-actin·S1 by 25 µM ATP. The observed transients were similar to those in *A* and a fit of the ADP dependence of the k_obs_ values to a hyperbola gave a half maximum inhibition (K’_5_) of 250 µM. ***D.*** The ADP inhibition of the ATP induced dissociation of pyrene-actin·S1 at a fixed ADP of 200 µM. The data for the fast phase in *B* is re-plotted with the k_obs_ values in the presence of ADP. The k_obs_ values are lower in all cases until saturation is reached with the k_max_ value being similar in the presence (498 s^-1^) and absence of ADP (430 s^-1^). This is the signature of ADP as a classical competitive inhibitor of ATP binding with the ADP equilibration being much faster than the maximum rate of ATP binding. ***E.*** Typical tryptophan fluorescence transient for ATP binding to 0.5 µM S1 in the absence of actin. The transients can be described by a single exponential with k_obs_ values of 9.4 and 36 s^-1^ for 10 and 40 µM ATP respectively and an amplitude of ~4% of the final fluorescence signal. ***F.*** A plot of the [ATP] dependence of the k_obs_ values shown fitted to a hyperbolic function with k_max_ = (k_+3_ +k_-3_) = 150 s^-1^.

**Figure S4:** **Myosin sequence alignments.**  Sequence alignment using CLUSTALW of the α- and β-isoforms from mouse, rat and human together with the bovine β-isoform, show the high sequence conservation between the two isoform groups. Coloring represents residue conservation with conserved residues in dark blue, semi-conserved residues in light-blue and non-conserved residues in white.

A

B

180°

A

B

**Figure S5: Homology model of human β-myosin S1 showing non-conserved residues (space filling) compared with α-myosin suggest alterations in the communication between the actin- and nucleotide- binding site.** An overview of the myosin S1 (bottom) is enlarged in panel A and rotated 180° in panel B. Residues that interact directly with actin are indicated in yellow (A^423^S, X^421^Y, N^416^S and Q^595^E) and are close to the cardiomyopathy loop (HCM-loop, dark-green) and loop 2 (light-green). Residues I^303^V, I^313^V, T^318^V and T^319^S (dark-blue) are linked to switch-1 via variable residue D^282^N (light-blue). Loop 1 is shown green, helix K is shown in red and helix L in purple. The latter contains residues N^347^A, S^348^G and M^349^V, close to variable residues L^617^T and S^618^A (red) which are near loop 2. The location of the nucleotide (space-filling model in variable colors), together with the P-loop (purple) and switch 1 (SW-1, green) is also shown.
